# Supplementary material for: End-to-end reproducible AI pipelines in radiology using the cloud
Source: Nat Commun. 2024 Aug 13;15:6931. doi: 10.1038/s41467-024-51202-2 (PMC11322541; doi:10.1038/s41467-024-51202-2)
Supplement: Supplementary file 1 — Supplementary Information [file 41467_2024_51202_MOESM1_ESM.pdf]

Supplementary Information for "End-to-End Reproducible AI Pipelines in Radiology Using the Cloud"

Authors | Dennis Bontempi[1,2,3], Leonard Nuernberg[1,2,3], Suraj Pai[1,2,3], Deepa Krishnaswamy[4], Vamsi Thiriveedhi[4], Ahmed Hosny[1,3], Raymond H Mak[1,3], Keyvan Farahani[5], Ron Kikinis[4], Andrey Fedorov[4], Hugo JWL Aerts[1,2,3]

Affiliations

[1] Artificial Intelligence in Medicine (AIM) Program, Mass General Brigham, Harvard Medical School, Boston, United States of America.

[2] Radiology and Nuclear Medicine, CARIM & GROW, Maastricht University, Maastricht, The Netherlands.

[3] Department of Radiation Oncology, Dana-Farber Cancer Institute, Harvard Medical School, Boston, United States of America.

[4] Department of Radiology, Brigham and Women's Hospital, Harvard Medical School, Boston, United States of America.

[5] National Heart, Lung, and Blood Institute and National Cancer Institute, Bethesda, Maryland

Supplementary Methods

Using Cloud-based Resources with Local Runtimes | Leveraging cloud-based computing to run the resources we share offers a democratizing, immediate, and easy solution - as it doesn't require setup of any kind nor readily available hardware. However, even when the primary intent is to run these pipelines on the cloud, we believe sharing resources developed with the proposed framework can bring multifaceted benefits to the community. Furthermore, by providing a way to run such pipelines locally, we eliminate the dependency on any third-party cloud-computing provider. To this end, we used publicly available Docker images[1] to reproduce the cloud-based pipelines on local nodes, achieving the same results. We provide the users with the documentation to replicate the setup as part of the project repository[2,3]. One of the major advantages of cloud-based pipelines remains the ease of integration: whether the user runs the pipelines locally or on the cloud, thanks to the shared Docker images, the underlying computational environment remains consistent. This means that transitioning between local and cloud processing, whether for overflow computational needs or collaborative projects, becomes seamless.

Practical Differences Compared to a Local Setup | In contrast with the section above, we want to provide an example of the steps a user (who we assume is familiar with the field and equipped with adequate computational hardware for the sake of simplicity) should follow if they want to set up everything necessary to replicate and extend Hosny et al.'s [4] pipeline on local hardware without the resources provided.

The first step in this process should be to set up a computational environment capable of running Hosny et al. - that is, python libraries and system dependencies. Next, the user must search for and download publicly available data (or private/institutional data) that suit the use case and can provide a reliable benchmark for the model and curate them. The user should then write the code to prepare the data for processing: as this is often the case, this requires the reimplementation of use-case-specific data preparation steps, often

omitted in the codebase (as for Hosny et al.), and has the potential to introduce inconsistencies in the results[5] (given the number of tools available to implement such steps often differs in some key aspects). Finally, the user must re-implement a result evaluation pipeline to reproduce or extend the findings of the original publication. This often relies, again, on the choice of a preferred tool among many that might require specific expertise to be run as intended.

None of the aforementioned challenges can, by their nature, be addressed by the reporting guidelines, checklists, or model registries (and zoos) that have been proposed in recent years to battle the reproducibility crisis. Furthermore, such challenges can be worsened by the fact that some of the steps involved can be very time-consuming (e.g., the selection of data to validate the pipeline) or demand very specific knowledge from different fields (e.g., the environment and model setup, the result analysis).

Specifics of the Google Colaboratory Instances

	CPU	Virtual cores	RAM	GPU	VRAM	Disk
**“Standard” CPU-only**	Intel Xeon (2.20GHz)	2	13GiB	-	-	116GiB
**“High RAM” CPU-only**	Intel Xeon (2.20GHz)	8	52GiB	-	-	243GiB
**“Free” GPU**	Intel Xeon (2.20GHz)	2	13GiB	T4	15GiB	84GiB
**“Pro” GPU**	Intel Xeon (2.00GHz	8	52GiB	V100	16GiB	180GiB

Supplementary Table 1 | Colab Instances specifics. Specifics of the different configurations of the Colab instances used for developing and testing the end-to-end pipelines.

IDC Queries | The Imaging Data Commons enables the user to query against any of the DICOM attributes in the collections hosted on the platform to build cohorts that can be recovered at any point in time. Here, we report the queries we used to build the datasets used in our study.

NSCLC-Radiomics

```
SELECT
    PatientID,
    StudyInstanceUID,
    SeriesInstanceUID,
    SOPInstanceUID,
    gcs_url
FROM
    `bigquery-public-data.idc_v16.dicom_all`
WHERE
    Modality IN ("CT",
        "RTSTRUCT")
    AND Source_DOI = "10.7937/K9/TCIA.2015.PF0M9REI"
ORDER BY
    PatientID
```

Supplementary Item 1 | NSCLC-Radiomics Query

The “SELECT” statement specifies five columns to be parsed from the specified table: the patient identifier, the study instance UID, the series instance UID, the Service-Object Pair (SOP) Instance UID, and the GCS URL. In this case, every row of the table resulting from the query will correspond to a DICOM file. The first four columns are DICOM attributes specific to the file, the series, or the study - while the last column will contain the URL to the corresponding DICOM file stored in Google Cloud Storage (GCS).

The “FROM” statement specifies the dataset and table from which to retrieve the data. In this case, the data will be fetched from the “dicom_all” table within the “idc_v16” dataset, part of BigQuery's public datasets (specifically, the bigquery-public-data project). Specifying the version of the IDC data to fetch makes this query reproducible at any point in time.

The “WHERE” statement specifies a filter applied to the query only to return rows where the DICOM attribute “Modality” has values "CT" (DICOM Computed Tomography Series) or "RTSTRUCT" (DICOM Radiation Therapy Structure Series). Furthermore, by specifying the DOI corresponding to the NSCLC-Radiomics, we ensure only patients belonging to the specific collections will be fetched.

Finally, the “ORDER” clause specifies that the resulting table should be sorted by the PatientID column. This will group the results by the patient identifier, making it easier to inspect the content of the table.

NSCLC-Radiogenomics

```
SELECT
  collection_id,
  PatientID,
  StudyInstanceUID,
  SeriesInstanceUID,
  SOPInstanceUID,
  Modality,
  gcs_url,
  InstanceNumber
FROM
  `bigquery-public-data.idc_v16.dicom_all`
WHERE
  (Modality IN ("CT", "SEG")) AND
  (collection_id = "nsclc_radiogenomics")
```

Supplementary Item 2 | NSCLC-Radiogenomics Query

The “SELECT” statement specifies five columns to be parsed from the specified table: in addition to the query in **Supplementary Item 1**, this query also parses the collection identifier, the modality, and the instance number. As for the other query, every row of the table resulting from the query will correspond to a DICOM file, with most columns containing DICOM attributes specific to the file (except for the GCS URL and the collection ID).

The “FROM” statement specifies the dataset and table from which to retrieve the data. In this case, the data will be fetched from the “dicom_all” table within the “idc_v16”

dataset, part of BigQuery's public datasets (specifically, the bigquery-public-data project). Specifying the version of the IDC data to fetch makes this query reproducible at any point in time.

The “WHERE” statement specifies a filter applied to the query only to return rows where the DICOM attribute “Modality” has values "CT" (DICOM Computed Tomography Series) or "SEG" (DICOM Segmentation object). Since NSCLC-Radiogenomics has both CT data and PET-CT data, this makes sure we are only fetching the DICOM files for the Computed Tomography. By specifying the DOI corresponding to the NSCLC-Radiomics, we ensure only patients belonging to the specific collections will be fetched.

Finally, the “ORDER” clause specifies that the resulting table should be sorted by the PatientID column. This will group the results by the patient identifier, making it easier to inspect the content of the table.

References

1. Google Artifact Registry - Google Colab Docker Images. https://console.cloud.google.com/artifacts/docker/colab-images/us/public/runtime.
2. *idc-radiomics-reproducibility*. (Github).
3. Dennis Bontempi. @AIM-Harvard @MHubAI. *ImagingDataCommons/idc-radiomics-reproducibility: Second release*. doi:10.5281/zenodo.10047767.
4. Hosny, A. *et al.* Deep learning for lung cancer prognostication: A retrospective multi-cohort radiomics study. *PLoS Med.* **15**, e1002711 (2018).
5. Mateus, P. *et al.* Image based prognosis in head and neck cancer using convolutional neural networks: a case study in reproducibility and optimization. *Sci. Rep.* **13**, 18176 (2023).
